# Pulp stones in unerupted teeth: a retrospective analysis using cone-beam computed tomography

**DOI:** 10.1186/s12903-024-04503-3

**Published:** 2024-06-21

**Authors:** Hassan Hamed Kaabi, Abdullah Mahmoud Riyahi, Abdullah Khalid Bakrman, Yazeed Ali Almutaw, Saleh Firas Alrumayyan, Nassr Saleh Al-Maflehi

**Affiliations:** 1 Department of Oral Medicine and Diagnostic Sciences, College of Dentistry, King Saud University, P.O. Box 60169, Riyadh, 11545 Saudi Arabia; 2 Division of Endodontic Dentistry, Department of Restorative Dental Sciences, College of Dentistry, King Saud University, P.O. Box 60169, Riyadh, 11545 Saudi Arabia; 3 College of Dentistry, King Saud University, P.O Box 60169, Riyadh, 11545 Saudi Arabia; 4 Division of Community Dentistry, Department of Periodontics and Community Dentistry, College of Dentistry, King Saud University, P.O Box 60169, Riyadh, 11545 Saudi Arabia

**Keywords:** Pulp stone, Pulp calcification, Unerupted teeth, Impacted teeth, Cone-beam computed tomography, CBCT, Systemic diseases, Saudi Arabia

## Abstract

**Background:**

A pulp stone is a calcified mass that develops in the dental pulp of any tooth. Despite many studies examining the relationship between pulp stone formation and non-oral factors, the methods used in these studies have been unable to explain the exact role of these factors alone as distinct from probable effects within the oral cavity environment. Considering that totally unerupted (impacted or developing) teeth are unexposed to the oral cavity’s environmental and functional conditions, they provide a more suitable material for studying the effects of these non-oral factors on pulp stone formation. This research study aimed to investigate pulp stones in unerupted teeth and the associated factors in a Saudi subpopulation.

**Methods:**

The study included 644 cone-beam computed tomography images, with 496 (50.9%) maxillary and 479 (49.1%) mandibular teeth. Of the investigated patients, 293 (45.5%) were men, and 351 (54.5%) were women. The age range was 15–76 years. A chi-square test was used to investigate the associations between pulp stones and age, gender, and history of systemic disease and chronic medication use.

**Results:**

Pulp stones in unerupted teeth were present in 24.2% of the examined dental jaws and 18.6% of the examined teeth. There was no statistically significant relationship between pulp stones and gender (*p* > 0.05). A significantly greater percentage of pulp stones were found with increasing age (*p* = 0.000). Additionally, a significantly increased number of pulp stones was observed in patients with systemic diseases and chronic medications (*p* < 0.05).

**Conclusions:**

The results support the idea that pulp stones can be present in any type of unerupted tooth. This study provides additional evidence of the increased incidence of pulp stones with age, systemic disease, and chronic medications.

## Introduction

A pulp stone is a discrete, calcified mass that can develop in the coronal or radicular pulp of any primary or permanent tooth. Pulp stones can be free, attached, or embedded into the dentin. Based on their histological structure, pulp stones are classified as true (dentin-like) or false calcifications; the former are made of dentin and lined by odontoblasts, and the latter are formed from mineralized, degenerated cells [1, 2]. Nodular and diffuse mineralization patterns of pulp stones are determined by the coronal cellular and radicular fibrous tissue content, respectively [3].

Several studies have reported a wide range of prevalence rates of pulp stones, ranging from 8 to 90% [1]. At the local level, the prevalence of pulp stones in the Saudi population is greater, ranging between 4.6% and 98.3% [4–6]. The variation could be attributed to differences in the analysis methods utilized and population variation among the studies [6].

Pulp stone formation is subject to variations that are secondary to environmental conditions of the oral cavity, such as traumatic occlusion, dental caries, erosion, operative procedures, periodontal diseases, and orthodontic treatment. In addition, pulp stones have been correlated with several non-oral factors, including age, gender, systemic diseases, and chronic medications [1, 2, 7, 8]. Despite many studies examining the relationship between pulp stone formation and non-oral factors, the methods used in these studies have been unable to explain the exact role of these factors alone as distinct from probable effects within the oral cavity environment [6, 9, 10]. Considering that totally unerupted (impacted or developing) teeth are unexposed to the oral cavity’s environmental and functional conditions, they provide a more suitable material for studying the effects of these non-oral factors on pulp stone formation [7].

A limited number of studies have reported the presence of pulp stones in totally unerupted teeth, whether impacted or developing. In 1986, Nitzan et al. found pulp stones in 56% of the studied impacted teeth; however, the study was limited to the effect of aging on only extracted canines [7]. A case report suggested an idiopathic rather than a pathologic etiology for pulp stone presence in developing, unerupted, permanent teeth [11].

Two-dimensional radiographs (e.g., periapical, bitewing, panorama) are commonly used for pulp stone examination. However, these methods are limited to detecting stones larger than 200 μm [12]. Another obvious limitation of these imaging modalities is the lack of a third dimension. Currently, cone-beam computed tomography (CBCT) is utilized for complex procedures in dental practice where regular radiographs are insufficient. The CBCT technique involves creating holistic, 3D scans with additional information for accurate diagnostic and treatment requirements [13, 14]. The geometrical representation provided by CBCT prevents the superimposition of neighboring structures [15]. Therefore, CBCT images are useful and accurate for studying pulp stone prevalence and distribution.

Research on the presence of pulp stones in unerupted teeth is scarce [7, 11]. Unerupted teeth are usually excluded from the analysis in research studies investigating pulp stones, which may underestimate the effects of non-oral factors. Pulp stones in unerupted teeth may provide useful information on the association with such factors, including age, gender, and chronic medications. Also, they may serve in diagnosing underlying systemic diseases more accurately than erupted teeth, which are exposed to several factors in the oral environment. To our knowledge, no previous study has utilized CBCT on a relatively large sample to assess the presence of pulp stones exclusively in unerupted teeth. This retrospective study aimed to use CBCT images to meticulously assess the presence of pulp stones in completely unerupted teeth in a Saudi population. The associations between pulp stones and age, gender, systemic diseases, and chronic medications were also investigated.

## Methods

### Ethical approval

The current retrospective study was ethically reviewed and approved by the Institutional Review Board of the Research Ethics Committee of King Saud University (IRB No. E-22-7460). It was also registered at the College of Dentistry Research Center (CDRC No. FR0663). The data used in this retrospective study were obtained from the King Saud University Dental Hospital’s database without risk to human subjects. Informed consent was not required considering the retrospective nature of the study.

### Inclusion and exclusion criteria

Using the statistical tool (G* POWER 3.1.9), the sample size was determined to be 644 cases following the criteria of alpha cut-off of 5% (0.05) with an effect size of 0.16 and power of 0.90. All CBCT images of the upper and lower jaws between October 2023 and April 2017 (*n* = 9243) were screened for the presence of unerupted teeth. According to the inclusion and exclusion criteria, the study included 644 CBCT images (322 upper and 322 lower jaws). The study included all permanent teeth that were impacted or developing, both of which were totally unexposed to the oral environment [7, 11, 16]. The present study included clear CBCT images of complete upper or lower jaws for Saudi patients aged 15 years or older with at least one unerupted tooth with half of its root length formed. Poor-quality CBCT scans and unerupted teeth with pathology were excluded.

### Image analysis

**Fig. 1:**
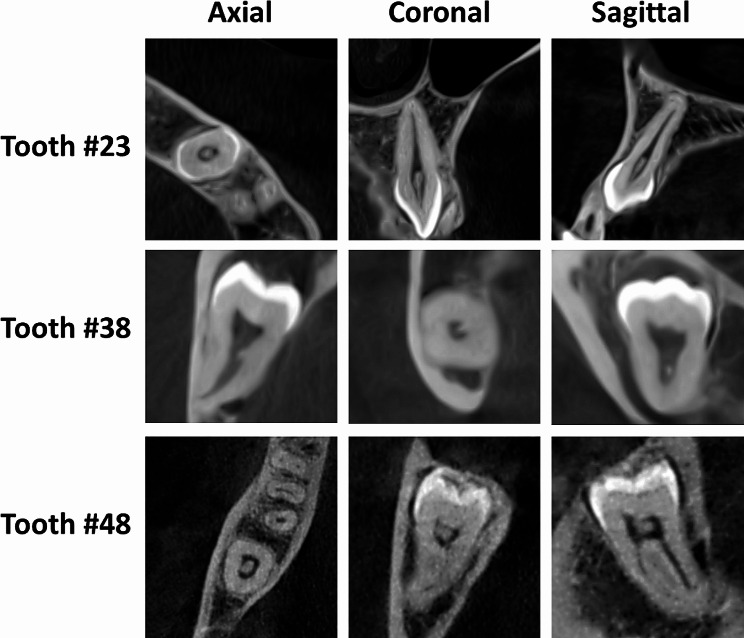
CBCT images showing dental pulp stones in different positions of different unerupted teeth. Teeth #23 and #48 are vertically impacted, whereas tooth #38 is horizontally impacted

Images of the examined cases were acquired using a ProMax 3D Max device (Planmeca, Helsinki, Finland). The tube voltage, tube current, and scanning time were 90 kV, 11 mA and 12 s, respectively. The voxel size was 150–200 μm, and the field of view was 12 cm × 10 cm. The images were analyzed using Romexis imaging software (Planmeca, Helsinki, Finland) and viewed on a 34-inch LED monitor. Each case was analyzed by two trained observers who performed a kappa test and who scored intra- and interexaminer reliability values of 0.92 and 0.87, respectively. While screening the cases, the observers had access to the CBCT images, which only showed the patient file number, without access to any other data. Once the CBCT examination was completed for all cases, the patient data were accessed and mapped to CBCT cases. Pulp stones were confirmed in the examined teeth if at least one discrete, radiopaque mass was observed within the coronal or radicular pulp space in all three planes (coronal, sagittal and axial) (Fig. 1). The image brightness and contrast were adjusted for optimal analysis, and the evaluation was performed in a dark room. Non-oral factors, including age, gender, systemic diseases, and chronic medications, were recorded from the patient’s hospital digital files. A decade-based distribution of age groups was used (15–25, 26–35, 36–45, > 45).

### Statistical analysis

Descriptive statistics, frequencies, and percentages are presented. For statistical inference, the chi-square test was utilized to evaluate the associations between categorical variables. The significance level was set at 0.05; any test result with a *p* value less than 0.05 was considered significant. The Statistical Package for the Social Sciences (SPSS) version 26 (IBM, Armonk, NY, USA) was used to analyze the collected data.

## Results

In total, 644 CBCT images were analyzed for pulp stones in unerupted teeth. The study included 322 maxillary and 322 mandibular arches, of which 293 (45.5%) were men and 351 (54.5%) were women. The age range for the examined cases was 15–76 years, with a mean age of 28.82 years (± 10.9).

Table 1 presents the descriptive characteristics and general distribution of pulp stones. Among the 644 dental arches studied, 156 (24.2%) had at least one unerupted tooth with a pulp stone. Moreover, pulp stones were found in 181 (18.6%) of the total unerupted teeth examined (*n* = 975). Multiple factors, including gender, age, systemic diseases, and chronic medication, were further examined to explore their possible associations with pulp stone formation in unerupted teeth.

**Table 1:** Descriptive characteristics (arch-wise)

	Examined	With stone	No stone
	*N* (%)	*N* (%)	*N* (%)
**Dental arches**	644	156 (24.2)	488 (75.8)
Maxilla	322 (50)	84 (26.1)	238 (73.9)
Mandible	322 (50)	72 (22.4)	250 (77.6)
**Gender**			
Male	293 (45.5)	72 (24.6)	221 (75.4)
Female	351 (54.5)	84 (23.9)	267 (76.1)
**Age (years)**			
15–25	318 (49.4)	64 (20.1)	254 (79.9)
26–35	178 (27.6)	32 (18)	146 (82)
36–45	90 (14)	35 (38.9)	55 (61.1)
> 45	58 (9)	25 (43.1)	33 (56.9)
**Systemic diseases**			
Yes	106 (16.5)	39 (36.8)	67 (63.2)
No	538 (83.5)	117 (21.7)	421 (78.3)
**Chronic medications**			
Yes	92 (14.3)	29 (31.5)	63 (86.5)
No	552 (85.7)	127 (23)	425 (77)
**Total teeth**	975	181 (18.6)	794 (81.4)
Maxillary teeth	496 (50.9)	94 (19)	402 (81)
Mandibular teeth	479 (49.1)	87 (18.2)	392 (81.8)
**Tooth type**			
Incisor	15 (1.5)	3 (1.7)	12 (98.3)
Canine	186 (19.1)	32 (17.7)	154 (82.3)
Premolars	54 (5.5)	2 (1.1)	52 (98.9)
1st and 2nd molars	18 (1.8)	9 (5)	9 (95)
3rd molars	702 (72)	135 (74.6)	567 (25.4)

Table 2 shows no statistically significant relationship between pulp stones and gender. Table 3 describes the relationships between the age groups and the presence of pulp stones. The percentage of unerupted teeth with pulp stones increased significantly with age (*p* = 0.000). The relationship between pulp stone formation in unerupted teeth with systemic diseases and the use of chronic medications is shown in Table 4. There was a significant relationship between pulp stones, systemic diseases, and chronic medications (*p* < 0.05).

**Table 2:** Number of unerupted teeth with and without pulp stones according to gender

	Maxillary teeth				Mandibular teeth				Total examined teeth			
	Examined	With stone	No stone	*P*	Examined	With stone	No stone	*p*	Examined	With stone	No stone	*p*
	*N* (%)	*N* (%)	*N* (%)		*N* (%)	*N* (%)	*N* (%)		*N* (%)	*N* (%)	*N* (%)	
**Gender**												
Male	220 (44.4)	46 (20.9)	174 (79.1)	0.327	236 (49.3)	42 (17.8)	194 (82.2)	0.838	456 (46.8)	88 (19.3)	368 (80.7)	0.581
Female	276 (55.6)	48 (17.4)	228 (82.6)		243 (50.7)	45 (18.5)	198 (81.5)		519 (53.2)	93 (17.9)	426 (82.1)	

Significance level < 0.05; *p value*: *p* value (chi-square test)

**Table 3:** Number of unerupted teeth with and without pulp stones according to age

	Maxillary teeth				Mandibular teeth				Total examined teeth			
	Examined	With stone	No stone	*p*	Examined	With stone	No stone	*p*	Examined	With stone	No stone	*p*
	*N* (%)	*N* (%)	*N* (%)		*N* (%)	*N* (%)	*N* (%)		*N* (%)	*N* (%)	*N* (%)	
**Age (years)**												
15–25	295 (59.5)	44 (14.9)	251 (85.1)	0.000	226 (47.2)	29 (12.8)	197 (87.2)	0.000	521 (53.4)	73 (14)	448 (86)	0.000
26–35	112 (22.6)	17 (15.2)	95 (84.8)		146 (30.5)	21 (14.4)	125 (85.6)		258 (26.5)	38 (14.7)	220 (85.3)	
36–45	56 (11.3)	20 (35.7)	36 (64.3)		70 (14.6)	23 (32.9)	47 (67.1)		126 (12.9)	43 (34.1)	83 (65.9)	
< 45	33 (6.7)	13 (39.4)	20 (60.6)		37 (7.7)	14 (37.8)	23 (62.2)		70 (7.2)	27 (38.6)	43 (61.4)	

Significance level < 0.05; *p value*: *p* value (chi-square test)

**Table 4:** Number of unerupted teeth with and without pulp stones concerning systemic diseases and chronic medications

	Maxillary teeth			*p*	Mandibular teeth			*p*	Total examined teeth			*p*
	Examined *N* (%)	With stone *N* (%)	No stone *N* (%)		Examined *N* (%)	With stone *N* (%)	No stone *N* (%)		Examined *N* (%)	With stone *N* (%)	No stone *N* (%)	
**Systemic diseases**												
Yes	67 (13.5)	21 (31.3)	46 (68.7)	0.005	87 (18.2)	26 (29.9)	61 (70.1)	0.002	154 (15.8)	47 (30.5)	107 (69.5)	0.000
No	429 (86.5)	73 [17]	356 (83)		392 (81.8)	61 (15.6)	331 (84.4)		821 (84.2)	134 (16.3)	687 (83.7)	
**Chronic medication**												
Yes	52 (10.5)	16 (30.8)	36 (69.2)	0.022	76 (15.9)	20 (26.3)	56 (73.7)	0.044	128 (13.1)	36 (28.1)	92 (71.9)	0.003
No	444 (89.5)	78 (17.6)	366 (82.4)		403 (84.1)	67 (16.6)	336 (83.4)		847 (86.9)	145 (17.1)	702 (82.9)	

Significance level < 0.05; *p value*: *p* value (chi-square test)

## Discussion

The current study utilized CBCT images to evaluate the presence of pulp stones in unerupted teeth and their associated factors in a Saudi subpopulation. The examined factors included age, gender, systemic diseases, and chronic medication, which may be considered non-oral factors.

The current study used retrospective CBCT images to study the presence of dental pulp stones without exposing participants to unnecessary imaging. The CBCT technique involves creating holistic 3D scans with additional information for accurate diagnostic requirements. CBCT is advantageous to two-dimensional imaging, as the latter cannot detect pulp stones larger than 200 μm and are affected by the superimposition of neighboring structures (13–15).

The prevalence of dental pulp stones has been extensively investigated worldwide in different populations. This investigation revealed several possible factors associated with pulp stone formation. These factors include mastication, bruxism, caries, attrition, abrasion, dental procedures and materials, periodontal diseases, age, gender, dental arch, tooth type, systemic diseases, medications, and genetic predisposition. However, discrepancies in the results between pulp stone studies are apparent in terms of prevalence and risk factors [1, 2, 4, 8, 10, 17]. The erupted teeth are exposed to many confounding factors in the oral cavity that might not be covered by the same studies, which may over/underestimate the possible effects of the examined factors [18]. Unerupted teeth are defined as teeth that are not exposed to the oral cavity under different conditions. Therefore, they constitute suitable material for studying pulp stones and related factors other than those of the oral cavity. This may eliminate the influence of the environmental conditions in the oral cavity and provide a more appropriate estimate of the association between the exposure and the outcome [7].

The screened CBCT images in the present study were selected for individuals aged 15 years and older to investigate unerupted teeth. The minimum age of alveolar emergence of third molar teeth is 14.6 [19]. Further, all permanent teeth (excluding third molars) should normally erupt by this age [20]. Therefore, at age 15, any permeant teeth (excluding third molars) not emerging into the oral cavity were considered impacted. The third molars that were unexposed to the oral cavity and fulfilled the inclusion/exclusion criteria were included as unerupted regardless of whether they were developing or impacted.

The current study showed that pulp stones are not limited to erupted teeth that are exposed to the oral environment. Pulp stones in unerupted teeth were present in 24.2% of the examined dental jaws and 18.6% of the examined teeth. Only a limited number of researchers have studied the presence of pulp stones in totally unerupted teeth, whether impacted or developing. In 1986, Nitzan et al. found pulp stones in 56% of histologically analyzed impacted teeth [7]. Another study used periapical and bitewing radiographs and reported a lower incidence (9%) of pulp stones associated with impacted teeth [21]. Moreover, pulp stones in developing, unerupted, permanent teeth were observed in panoramic radiographs and were considered idiopathic rather than pathological [11]. The incidence of pulp stones significantly varies among studies, reflecting the different methods used to identify pulp stones. Among the methods used, the highest incidence of pulp stones is observed by histological examination, followed by CBCT and two-dimensional radiographs [6, 22]. The present study evaluated a relatively large sample size of exclusively unerupted teeth in three dimensions using CBCT; to our knowledge, no previous research has examined this topic.

Earlier studies demonstrated that women are more likely to experience pulp calcification due to bruxism [23]. However, other studies have reported a higher incidence in men, possibly due to the increased incidence of pulpal inflammation [24]. In agreement with other reports [6, 13], the findings of the present study demonstrated no significant association between the presence of pulp stones and gender. Since the examined teeth were unerupted, they were not exposed to the abovementioned risk factors that put one gender at a greater risk of pulp stone formation.

Our findings revealed higher percentages of unerupted teeth with pulp stones with increasing age, which agrees with earlier studies [6, 25]. A possible explanation may be the increased fat and fibrous deposition in the dental pulp that occurs with aging, which serves as a nidus for stone formation [26, 27]. However, this finding contradicts a study of impacted teeth that found a constant incidence of pulp stones for all different age groups, suggesting no relationship with aging. The study proposed that the presence of pulp stones in the young age group was a normal variation similar to individual biological characteristics such as cutaneous nevi and tori. Additionally, these findings indicate that dental pulp stones might develop in response to the eruptive efforts of impacted teeth. It should be noted that this study used only impacted canines [7]. In contrast, all types of unerupted teeth were included in the present study, which could be a possible reason for the inconsistency.

An increased prevalence of pulp stones was reported in medically compromised patients compared to healthy subjects [10, 28]. In agreement, the results of the present study showed a significant relationship between pulp stones and systemic diseases. The increased incidence of pulp stones in subjects with systemic conditions appears to be associated with systemic proinflammatory changes that result in pathological changes and dysregulation inside the pulp [28]. For example, a study attributed the higher prevalence of pulp stones in diabetic patients to obliterative endarteritis, in which the blood circulation of dental pulp is compromised, leading to calcification [10]. Other systemic diseases demonstrated to be risk factors for pulp stone development include cardiovascular diseases, diabetes mellitus, rheumatoid arthritis, tuberculosis, hypertension, and carcinoma [28]. The specific type of systemic diseases was not considered in the current study; this area of research is important in future studies.

It is worth mentioning that our study revealed a significant relationship between medications and pulp stones. Medications have relatively similar systemic effects to those of chronic diseases, which results in pathological changes in the dental pulp [28]. The use of various medications, such as statins and glucocorticoids, was reported to be a risk factor for pulp stone development. In contrast, other medication types, such as metformin, thyroxine, and gliclazide, did not show any association with pulp calcification [29]. The current research compared chronic users and nonusers of medications without investigating the specific type of medication. Therefore, more intensive studies associating specific types of medications and pulp stones are recommended.

This study has several limitations. Considering the retrospective nature of the present study, causal pathways between variables cannot be established. However, a significant relationship was found between pulp stones and age, systemic diseases, and chronic medications. Although the study minimized confounding factors by using unerupted teeth in comparison to erupted teeth, the examined teeth are likely to be affected by a variety of endogenous factors. CBCT provides valuable information for pulp stone analysis; however, the number might be underestimated compared to the histological analysis. Additionally, the collected data were limited to one dental hospital.

## Conclusions

In our study, there was an association between age, systemic disease, and chronic medication use and the presence of pulp stones in unerupted teeth. Nevertheless, no association was found between pulp stones and gender. The current study’s findings support the idea that pulp stones can be present in any unerupted tooth not exposed to oral conditions. Moreover, this study provides additional evidence of the increased incidence of pulp stones with age, systemic disease, and chronic medication use. Unerupted teeth may provide useful information for studying factors associated with pulp stones, such as a predictor of underlying systemic diseases. CBCT provides an accurate tool for detecting pulp stones with three-dimensional anatomical details. Future studies should consider unerupted teeth in pulp stone studies. Dental professionals should be aware of the presence of pulp stones in unerupted teeth and the associated factors.

## Data Availability

The data will be made available upon reasonable request to the corresponding author (Hassan Kaabi).

## Abbreviations

CBCT: Cone-beam computed tomography
IRB: Institutional Review Board
CDRC: College of Dentistry Research Center
SPSS: Statistical Package for the Social Sciences
IBM: International Business Machines Corporation
3D: Three-dimensional
kV: Kilovolt
mA: Milliampere
s: Second
µm: Micrometer
cm: Centimeter
LED: Light-emitting diode
USA: United States of America
NY: New York
